# Synaptic Effects of Munc18-1 Alternative Splicing in Excitatory Hippocampal Neurons

**DOI:** 10.1371/journal.pone.0138950

**Published:** 2015-09-25

**Authors:** Marieke Meijer, Tony Cijsouw, Ruud F. Toonen, Matthijs Verhage

**Affiliations:** Department of Functional Genomics, Center for Neurogenomics and Cognitive Research (CNCR), Neuroscience Campus Amsterdam (NCA), VU University Amsterdam and VU University Medical Center, Amsterdam, Netherlands; University of Edinburgh, UNITED KINGDOM

## Abstract

The *munc18-1* gene encodes two splice-variants that vary at the C-terminus of the protein and are expressed at different levels in different regions of the adult mammalian brain. Here, we investigated the expression pattern of these splice variants within the brainstem and tested whether they are functionally different. Munc18-1a is expressed in specific nuclei of the brainstem including the LRN, VII and SOC, while Munc18-1b expression is relatively low/absent in these regions. Furthermore, Munc18-1a is the major splice variant in the Calyx of Held. Synaptic transmission was analyzed in autaptic hippocampal *munc18-1* KO neurons re-expressing either Munc18-1a or Munc18-1b. The two splice variants supported synaptic transmission to a similar extent, but Munc18-1b was slightly more potent in sustaining synchronous release during high frequency stimulation. Our data suggest that alternative splicing of Munc18-1 support synaptic transmission to a similar extent, but could modulate presynaptic short-term plasticity.

## Introduction

Alterative splicing is a major regulatory mechanism estimated to occur in 95% of human multi-exon genes [1, 2]. Many examples of alternative splicing with functional consequences are found in neuron specific proteins, e.g. calcium channels and different classes of receptors, including glutamate and GABA receptors, altering the localization and response kinetics of these proteins [3]. A striking example is the large number of isoforms created by alternative splicing of the adhesion molecules neuroligin and neurexin. The synapse-inducing action of the neuroligin-neurexin complex toward glutamatergic or GABA-ergic synapses is determined by selective interactions between splice-variants [4]. Alternative splicing thus regulates synaptic transmission by contributing to synapse specificity. Another important way to regulate synaptic transmission is by modulating the synaptic vesicle release machinery, with the SNARE proteins syntaxin, SNAP-25 and synaptobrevin at its core [5, 6]. SNAP-25 undergoes alternative splicing generating two variants that differ in their N-terminal SNARE domain and their capacity to support primed vesicles, thereby modulating synaptic strength [7]. Alternative splicing of SNAP-25 is temporally regulated and might underlie the shift to a larger primed vesicle pool during synapse maturation.

Another essential component of the synaptic vesicle release machinery is the SM-protein Munc18-1 [6, 8, 9]. Alternative splicing of the mammalian Munc18-1 gene leads to two closely related gene products, which differ due to the inclusion (leading to Munc18-1a with a length of 603 aa) or exclusion (leading to Munc18-1b with a length of 594 aa) of a nucleotide sequence of 110 base pairs at the COOH terminus [10]. Exclusion of the sequence leads to a frame shift, which generates the C-terminus of Munc18-1b that is otherwise part of the un-translated sequence of Munc18-1a [10]. The two variants are expressed at different levels in different areas of the adult mammalian brain, with Munc18-1a being most abundant in brain stem and Munc18-1b in striatum, hippocampus, olfactory bulb, and cerebral cortex, while both are expressed at similar levels in cerebellum [10]. Since other localisation studies used probes that could not distinguish between both splice variants, little is known about their cell-type specificity or temporal expression [11]. Different types of neurons might express different splice variants to obtain a unique release machinery, or alternative splicing might be regulated within the same neurons to modulate synaptic transmission. Several splice variant unique phosphorylation sites have been previously identified in brain lysate using high-throughput screens [12–15] (search was conducted using PhosphositePlus [16]). However, whether the difference between Munc18-1a and Munc18-1b at protein level translates to a functionally different protein is unknown.

Here, we investigated whether alternative splicing of the Munc18-1 gene constitutes a regulatory mechanism of synaptic vesicle release. For this purpose, we expressed a single splice variant in cultured autaptic *munc18-1* null neurons, thus creating neurons that rely solely on Munc18-1a or Munc18-1b for synaptic transmission. Whole-cell recordings from these neurons suggest minor functional differences between the two splice variants, with Munc18-1a expressing neurons displaying faster synaptic depression during high frequency stimulation. These results suggest that alternative splicing of Munc18-1 can regulate short-term synaptic plasticity (STP).

## Materials and Methods

### Animals

Munc18-1 deficient mice were generated as described previously [8]. Munc18-1 null mutant mice are stillborn and can be easily distinguished from wild-type or heterozygous littermates. E18 embryos were obtained by caesarean section of pregnant females from timed matings of heterozygous mice. Newborn P0-P1 pups from pregnant female Wistar rats were used for glia preparations. Munc18-1-Venus knock-in (KI) mice were generated as described previously [17]. Munc18-1-Venus KI mice have the stop codon of exon 20 of *munc18-1* replaced with Venus cDNA. Therefore, only splice variant Munc18-1b expresses fluorescent Venus (fused to the C-terminus of Munc18-1b, Munc18-1b-Venus). In splice variant Munc18-1a, by inclusion of exon 19 harbouring an alternative stop codon, exon 20 and Venus become 3’-untranslated region and are not expressed. Animals were housed and bred according to institutional, Dutch and U.S. governmental guidelines. These studies were approved by the institutional ethical committee of the VU University (Protocol FGA 11–03).

### Constructs

M18-1a and M18-1b were cloned into pIRES_2_EGFP (Clontech) and verified by sequencing. Constructs were then subcloned into pLenti vectors, and viral particles were produced as described [18]. Transduction efficiencies were assessed on HEK293T cells using a concentration range, and were taken into account when viruses were applied to neuronal cultures. For this purpose, HEK293T cells were infected with lentivirus 1 day after plating in DMEM medium containing 10% FCS (Gibco) and 1% penicillin/streptomycin (Gibco). Upon reaching confluence, infected cells were counted based on EGFP expression.

### Dissociated neuronal cultures

Neuronal cultured were prepared according to [19]. Hippocampi from *munc18-1* null mice were collected in ice-cold Hanks Buffered Salt Solution (HBSS; Sigma) buffered with 7mM HEPES (Invitrogen). After removal of the meninges, neurons were incubated in Hanks-HEPES containing 0.25% trypsin (from 10x stock, Invitrogen) for 20 minutes at 37°C. After washing, neurons were triturated using a fire-polished Pasteur pipette and counted in a Fuchs-Rosenthal chamber. Neurons were plated in pre-warmed Neurobasal medium supplemented with 2% B-27, 1.8% HEPES, 0.25% glutamax and 0.1% Pen/Strep (all Invitrogen) and infected with lentiviral particles encoding Munc18-1 splice variants several hours after plating.

To achieve autaptic cultures, hippocampal *munc18-1* null neurons were plated on micro-islands of rat glia at a density of 6K per well in a 12-well plate. To generate these micro-islands, glass coverslips (Menzel) were etched in 1M HCl for at least two hours and neutralized with 1M NaOH for maximum one hour, washed thoroughly with MilliQ water and washed once with 70% ethanol. Coverslips were stored in 96% ethanol and coated with agarose type II-A (0.0015% in H_2_0, Sigma) prior to microdot application. Coating was done by spreading a thin layer of agarose solution (heated in microwave and kept at 55°C during use) with a cotton swab over the entire coverslip. Microdots were created using a custom made rubber stamp (dot diameter 250μm) to apply solution consisting of 0.1mg/ml poly-D-lysine (Sigma), 0.7mg/ml rat tail collagen (BD Biosciences) and 10mM acetic acid (Sigma) by stamping from a wet filter paper (3mm cellulose chromatography paper (Whatman)). Coverslips were UV-sterilized for 20 minutes before further use. Astrocytes were plated at 6-8K/well in pre-warmed DMEM medium (Invitrogen) supplemented with 10% fetal calf serum (FCS), 1% nonessential amino acids (NAA) and 1% penicillin/streptomycin (all Gibco).

For confocal microscopy experiments, hippocampal neurons were plated at 50K/well in 12-well plates containing glass coverslips disinfected with 96% ethanol and coated with 0.5 milli-percent poly-L-ornithine (Sigma) and 2μg/ml laminin (Sigma) in PBS overnight and thoroughly washed.

### Electrophysiological recordings

Autaptic cultures of *munc18-1* null neurons were grown for 13–18 days before measuring as reported in [19]. Whole-cell voltage-clamp recordings (Vm = -70mV) were performed at room temperature with borosilicate glass pipettes (2.5–4.5 mOhm) filled with 125mM K^+^-gluconic acid, 10mM NaCl, 4.6mM MgCl_2_, 15mM creatine phosphate, 10U/ml phosphocreatine kinase and 1mM EGTA (pH 7.30). External solution contained the following (in mM):10 HEPES, 10 Glucose, 140 NaCl, 2.4 KCl, 4 MgCl_2_ and 4 CaCl_2_ (pH = 7.30, 300 mOsmol). Inhibitory neurons were identified and excluded based on the decay of postsynaptic currents (as described in [20]). Recording were acquired with an Axopatch 200A amplifier (Molecular Devices), Digidata 1322A and Clampex 9.0 software (Molecular Devices). After whole cell mode was established, only cells with an access resistance of <12 MΩ, leak current of <500 pA and EPSC size > 300pA were accepted for analysis. EPSCs were elicited by a 0.5ms depolarization to 30 mV. RRP size was assessed by back-extrapolation (fitted through the last 40 pulses) of the cumulative synchronous charge elicited by a RRP depleting stimulation train (100 pulses at 40Hz) [21, 22]. Offline analysis was performed using Clampfit v9.0 (Axon Instruments), Mini Analysis Program v6.0 (synaptosoft) and custom-written software routines in Matlab 7.1 or R2009b (Mathworks).

### Immunochemistry

Hippocampal neurons were allowed to develop for 11 days before fixation. Cultures were fixed with 3.7% formaldehyde (Electron Microscopy Sciences). After washing with PBS, cells were permeated with 0.1% Triton X-100 for 5 min and incubated in 2% normal goat serum for 20 minutes to block nonspecific binding. Cells were incubated for 1 hr at room temperature in a primary antibody mixture of monoclonal mouse anti-VAMP (1:1000, SySy) and polyclonal chicken anti-MAP2 (1:10000, Abcam) antibodies. After washing, cells were incubated for 1 hr at room temperature with secondary antibodies conjugated to Alexa dyes (1:1000, Molecular Probes) and washed again. Coverslips were mounted with DABCO-Mowiol (Invitrogen) and images were acquired with a confocal microscope (LSM 510, Carl Zeiss) using a 40x oil immersion objective (NA = 1.3) with 0.7x zoom at 1024 x 1024 pixels and averaged over two scans. Confocal settings were kept the same for all scans within an experiment. Neuronal morphology and protein levels were analyzed using automated image analysis routine [23].

For labeling of brain slices, P14-P21 WT and Munc18-1-Venus KI mice were perfused transcardially with 4% PFA in PBS, brains were removed and post-fixed in 4% PFA in PBS overnight. Subsequently, brains were submerged in 30% sucrose in PBS for 3 days prior to cryo-sectioning. Before incubation in blocking solution (5% normal goat serum, 2.5% BSA, 0.2% Triton X-100 in PBS) for 1 h, 35 μm cryo-sections were incubated in 1% H_2_O_2_ for 30 min and rinsed with PBS. Sections were incubated with primary antibody in blocking solution overnight at 4°C on a shaker. Primary antibodies used were rabbit polyclonal Munc18-1b (1:200, SySy) and goat polyclonal anti-Munc18-1a (EB06140, Everest Biotech). Cryosections were then washed 4 times in PBS and incubated with secondary antibody (Alexa Fluor, Invitrogen) diluted in blocking solution for 2 h on a shaker. Finally, cryosections were mounted in 2.5% DABCO (Invitrogen) in Mowiol on glass slides. All steps were performed at RT, unless otherwise stated. Images were acquired on a confocal microscope (LSM 510 Meta, Zeiss) with either a 10x air objective and 0.7x mechanical zoom or a 40x objective (1.3 NA) and 0.7x mechanical zoom.

### Immunoblotting

To characterize protein expression levels of Munc18-1, cultured *munc18-1* null cortical neurons expressing Munc18-1a or Munc18-1b were allowed to develop for 13 days after which they were washed in PBS and homogenized in Laemmli sample buffer consisting of 2% SDS, 10% glycerol, 0.26 M β-mercaptoethanol, 60 mM Tris-HCl pH 6.8, and 0.01% Bromophenol blue. Samples were separated on 10% SDS-Polyacrylamide gels using the standard SDS-PAGE technique. Proteins were transferred to nitrocellulose membranes using the standard western blot technique (1 hour at 350 mA). Blots were incubated in 2% powdered milk and 0.5% bovine serum albumin (BSA) in PBS containing 0.1% Tween-20 (PBS-tween) for 1 hr at 4°C, and incubated with primary antibodies (polyclonal rabbit anti-Munc18-1 directed against the common site S241 (1:1000, Phosphosolutions) and monoclonal mouse alpha-Tubulin (1:10000, SySy)) in PBS-tween for 1 hr at 4°C. After washing, blots were incubated with anti-rabbit or anti-mouse IRDye secondary antibodies (LI-COR) in PBS-tween for 1 hr at 4°C, and washed again. Blots were scanned with Odyssey Fc dual-mode imaging system (LI-COR) for 2 minutes in each channel (700 and 800 nm laser).

### Data analysis

Data are presented as mean values ± s.e.m., with n referring to the number of cells from each group unless stated otherwise. Statistical analysis was performed with Instat v3.05 software (GraphPad Software). Data samples were first tested for normality with the Kolmogorov and Smirnov test and for heterogeneity of variance with the method of Bartlett. If data allowed an unpaired t-test (with Welch correction if standard deviations are not equal) was used to determine statistical significance. If data failed to pass the normality test, the non-parametric Mann-Whitney U test was used. P-values below 0.05 are considered significant and are indicated as following: *P<0.05, ** P<0.01, *** P<0.001.

## Results

### Both splice variants support basal synaptic transmission in excitatory hippocampal neurons

**Fig 1:**
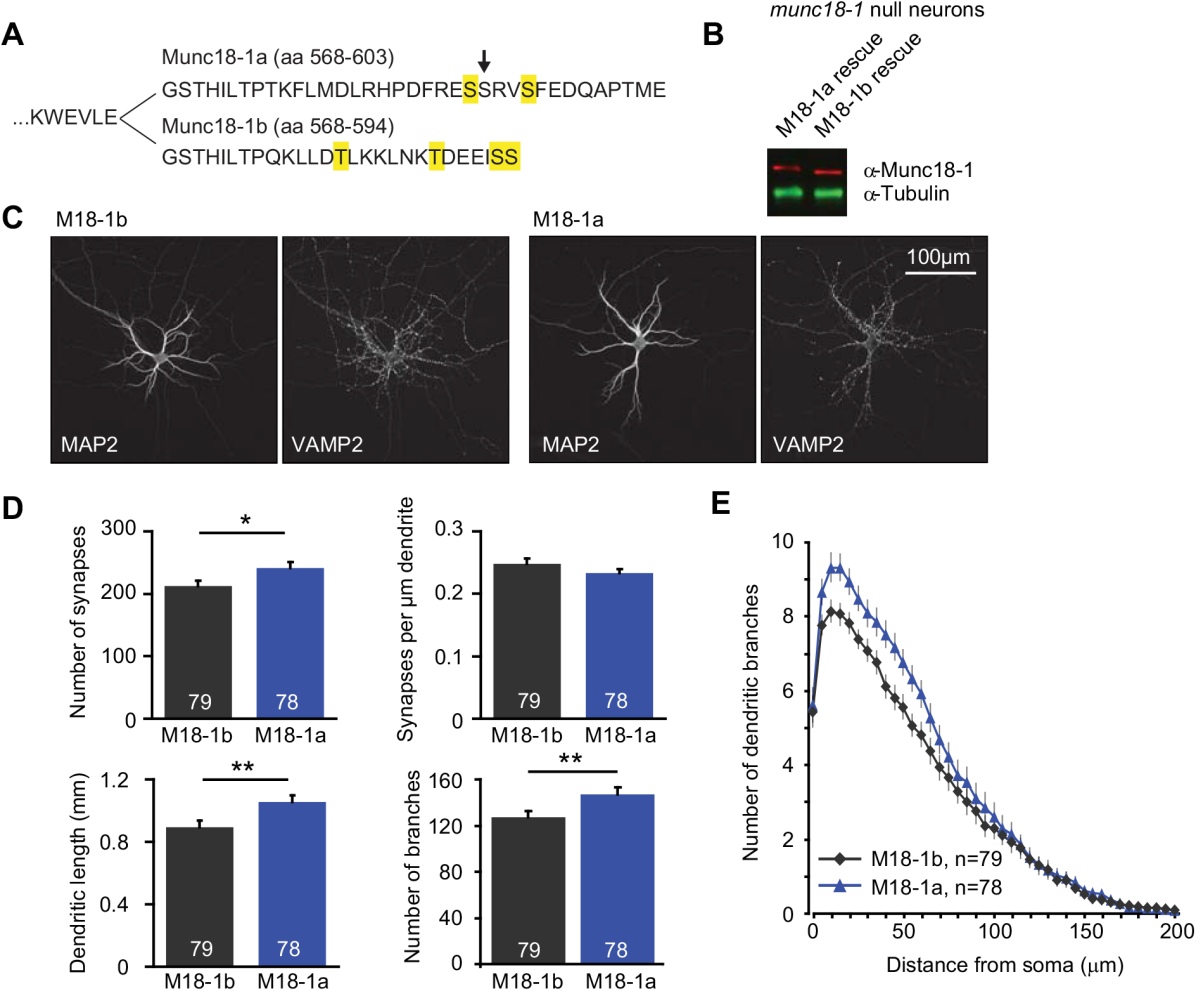
Morphology of hippocampal neurons expressing a single splice variant of Munc18-1. (A) Amino acid sequences of the C-terminus of mouse Munc18-1a and 1b starting at site 562 (adapted from [10]). Phosphorylation sites identified in high-throughput screens are highlighted (Fig 1b, M18-1a: S590, S594; M18-1b: T581, T588, S593, S594) [16]. Arrow points to a predicted CaMKII phosphorylation site in Munc18-1a identified using NetPhos 2.0 [40] and Phosida [38, 39]. (B) Representative immunoblot of Munc18-1 protein levels in rescued *munc18-1* null neurons. Neurons were analysed at DIV13 with western blot analysis using double immuno-fluorescent labelling for Munc18-1 (red) and α-Tubulin (green). (C-E) Munc18-1a or -1b was reintroduced using lentivirus in hippocampal *munc18-1* null neurons cultured in the absence of glia. (C) Examples of MAP2 (dendritic marker) and VAMP2 (synaptic marker) immunostainings to visualize neuronal morphology. Scale bar = 100 μm. (D) Quantification of neuronal morphology. Number of synapses (M18-1b: 212.1 ± 8.9 synapses, n = 79; M18-1a: 241.3 ± 10.0 synapses, n = 78, Unpaired t test, p = 0.0309). Synapse density (M18-1b: 0.248 ± 0.009 synapses/μm dendrite, n = 79; M18-1a: 0.233 ± 0.006 synapses/μm dendrite, n = 78, Mann-Whitney Test, p = 0.1466). Total dendritic length (M18-1b: 0.895 ± 0.040 mm, n = 79; M18-1a: 1.057 ± 0.041 mm, n = 78, Mann-Whitney Test, p = 0.009). Number of branches (M18-1b: 127.2 ± 5.6 branches, n = 79; M18-1a: 147.0 ± 6.0 branches, n = 78, Mann-Whitney Test, p = 0.0043). (E) Sholl analysis suggests that dendritic branching is more elaborate in Munc18-1a expressing neurons. Shown are of the number of dendrite crossings per ring. Rings are placed with increasing radius around the soma (increments of 5 μm).

Alternative splicing of Munc18-1 leads to two closely related splice variants that only differ at their C-terminus (Fig 1A). Although the splice variants differ by only ~4% of their amino acids, they might have evolved to meet specific cellular requirements. Therefore, we set out to test if the splice variants are functionally different in supporting synaptic transmission by expressing Munc18-1a or Munc18-1b in hippocampal neurons from *munc18-1* null mutant mice using Lentivirus. Both splice variants rescued the lethal phenotype of *munc18-1* null neurons [24]. Western blot analysis confirmed similar expression of Munc18-1a and -1b in rescued neurons (Fig 1B, note difference in Munc18-1 size). An automated image analysis routine [23] was used for morphological analysis of confocal images. Hippocampal neurons expressing Munc18-1a, which is normally not expressed in these neurons, had similar synapse density to neurons expressing Munc18-1b, but developed longer dendrites with more branches and had approximately 10% more synapses (Fig 1C–1E).

**Fig 2:**
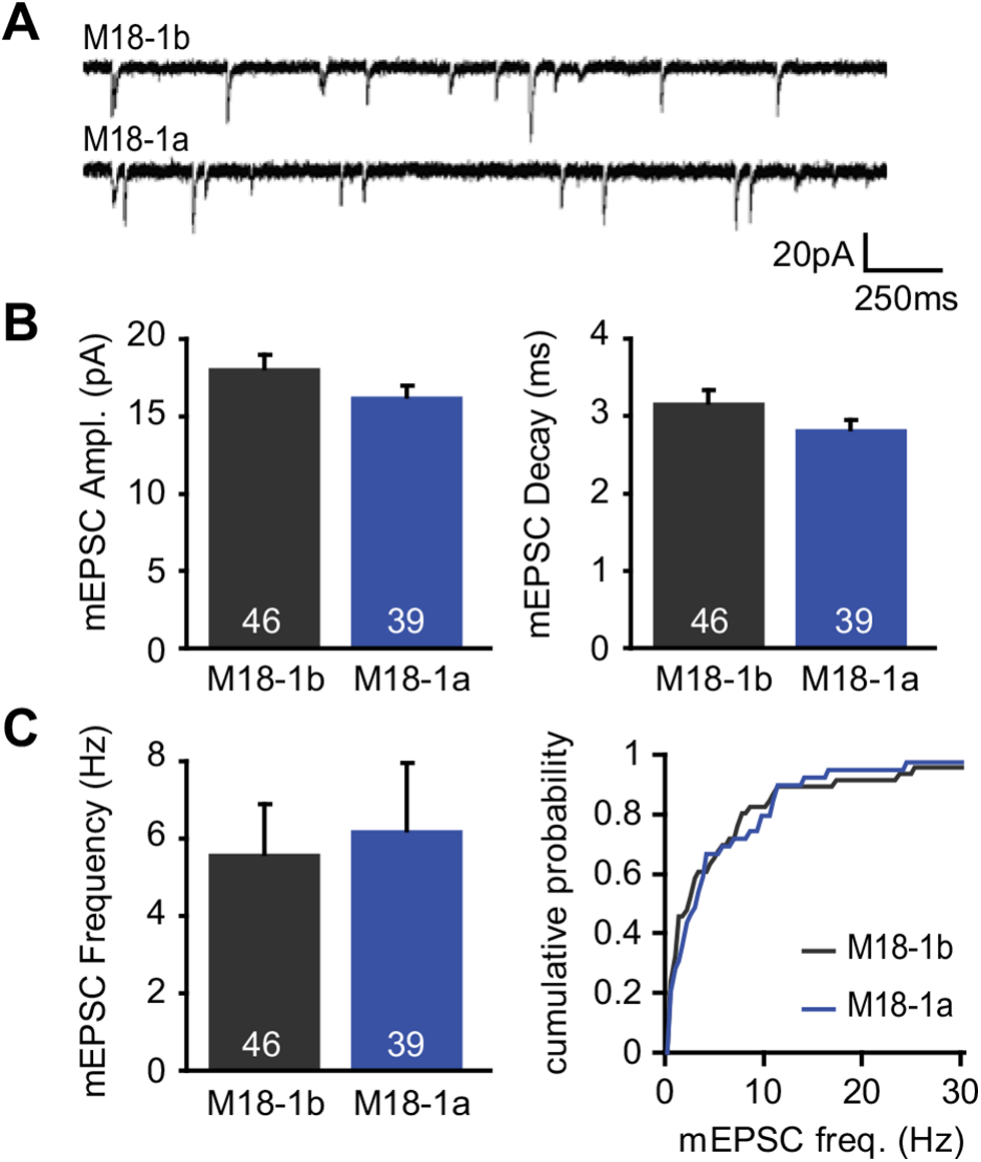
Alternative splicing of Munc18-1 does not affect spontaneous release. Munc18-1a or -1b was reintroduced using lentivirus in autaptic hippocampal *munc18-1* null neurons cultured on glia islands. (A) Example traces of spontaneous release of synaptic vesicles in the absence of stimulation. (B) Quantification of the mEPSC size and decay kinetics. mEPSC size (M18-1b: 18.11 ± 0.88 pA, n = 46; M18-1a: 16.29 ± 0.71 pA, n = 39, Unpaired t test with Welch correction, p = 0.11). mEPSC decay (M18-1b: 3.18 ± 0.15, n = 46; M18-1a: 2.84 ± 0.12, n = 39, Unpaired t test with Welch correction, p = 0.08). (C) Both splice variants support spontaneous release at similar rate. mEPSC frequency (M18-1b: 5.60 ± 1.29 Hz, n = 46; M18-1a: 6.21 ± 1.74 Hz, n = 39, Mann-Whitney Test, p = 0.5665.

Synaptic transmission was tested with whole-cell patch clamp measurements on autaptic neurons (neurons grown in isolation on glial islands) [25]. In the absence of stimulation, synaptic vesicles fuse spontaneously (Fig 2A), which enables the determination of quantal size and kinetics of these single fusion events (miniature excitatory postsynaptic currents or mEPSCs). No differences in size, decay kinetics or frequency of mEPSCs were found in neurons expressing Munc18-1b or Munc18-1a (Fig 2B and 2C).

**Fig 3:**
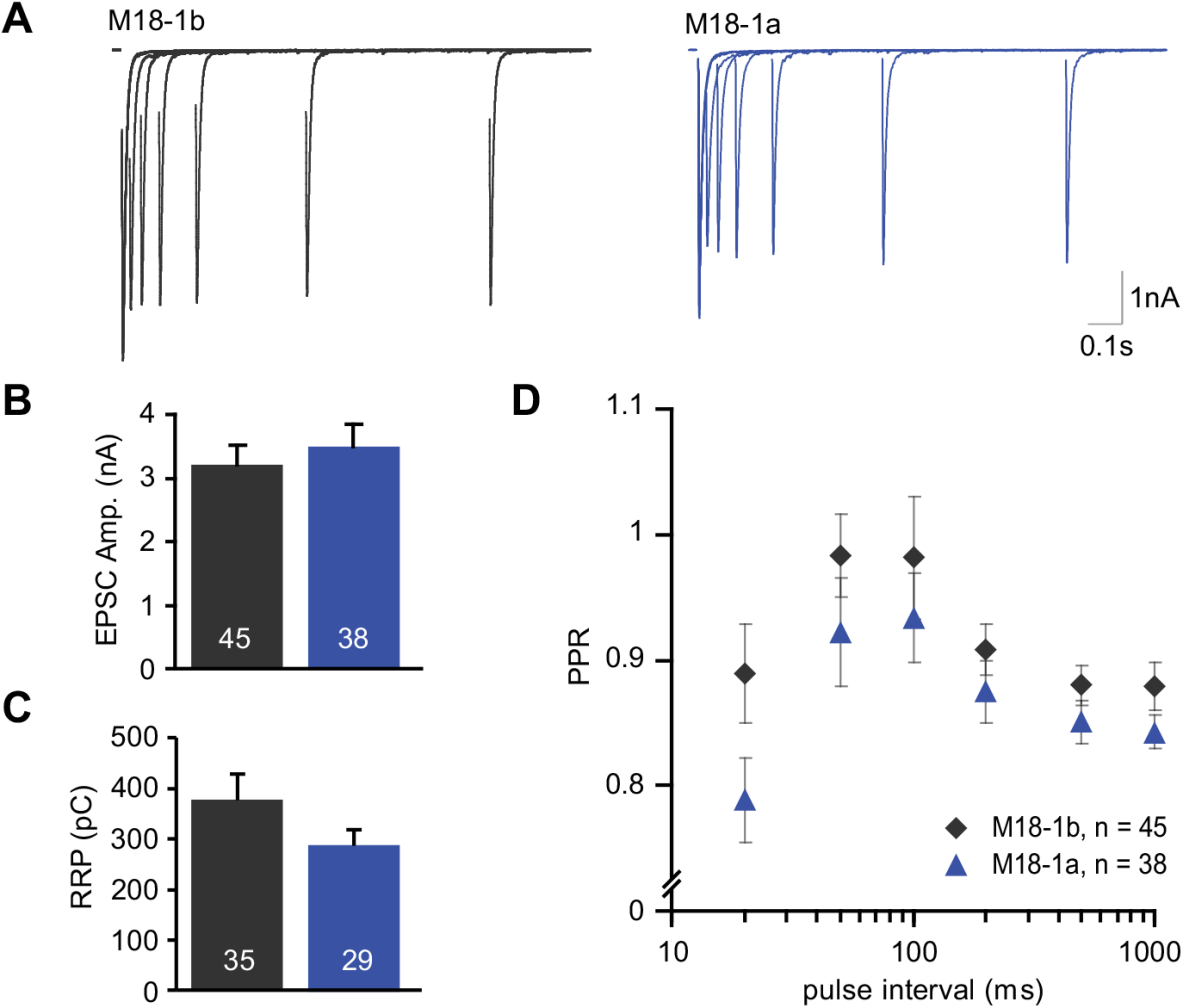
Alternative splicing of Munc18-1 does not affect basal evoked release. Munc18-1a or -1b was reintroduced using lentivirus in autaptic hippocampal *munc18-1* null neurons cultured on glia islands. (A) Typical examples of paired pulse traces, traces from different intervals are plotted superimposed. (B) Both splice variants support evoked release to the same extent. Initial EPSC size (M18-1b: 3.21 ± 0.31 nA, n = 45; M18-1a: 3.50 ± 0.35 nA, n = 38, Unpaired t test, p = 0.54). (C) Quantification of the RRP estimate derived from back-extrapolation of the cumulative charge released during a RRP depleting train (100 pulses at 40 Hz) (M18-1b: 377.8 ± 52.7 pC, n = 35; M18-1a: 287.3 ± 33.3, n = 29, Unpaired t test with Welch correction, p = 0.15). (D) Quantification of the paired pulse ratio for different intervals (20 ms interval: M18-1b, 0.89 ± 0.04 EPSC^2nd^/EPSC^1st^; M18-1a, 0.79 ± 0.03 EPSC^2nd^/EPSC^1st^, Unpaired t test, p = 0.060).

In addition, the peak current produced by synchronous evoked release was similar (Fig 3A and 3B), as was the size of the available vesicle pool (readily releasable pool (RRP), Fig 3C). The RRP was determined using a 40 Hz stimulation train, which completely depletes this pool (see materials and methods). Neurons were then subjected to a paired pulse protocol, in which the ratio between responses to two (paired) pulses given at various time intervals is used as a measure of release probability. A low release probability leads to a relatively large second response since most vesicles are still present, while a high release probability leads to depression of the second response due to the depletion of available vesicles. There were no differences in the paired pulse ratios (PPR), although a trend towards a lower PPR in neurons expressing Munc18-1a could be observed at the shortest interval tested (20ms, Fig 3A and 3D). This could be the result of a small increase in release probability in neurons expressing Munc18-1a. These results suggest that both Munc18-1 splice variants support basal spontaneous and evoked synaptic transmission in hippocampal neurons to the same extent.

### Neurons expressing Munc18-1b sustain synaptic transmission longer during periods of intense stimulation

**Fig 4:**
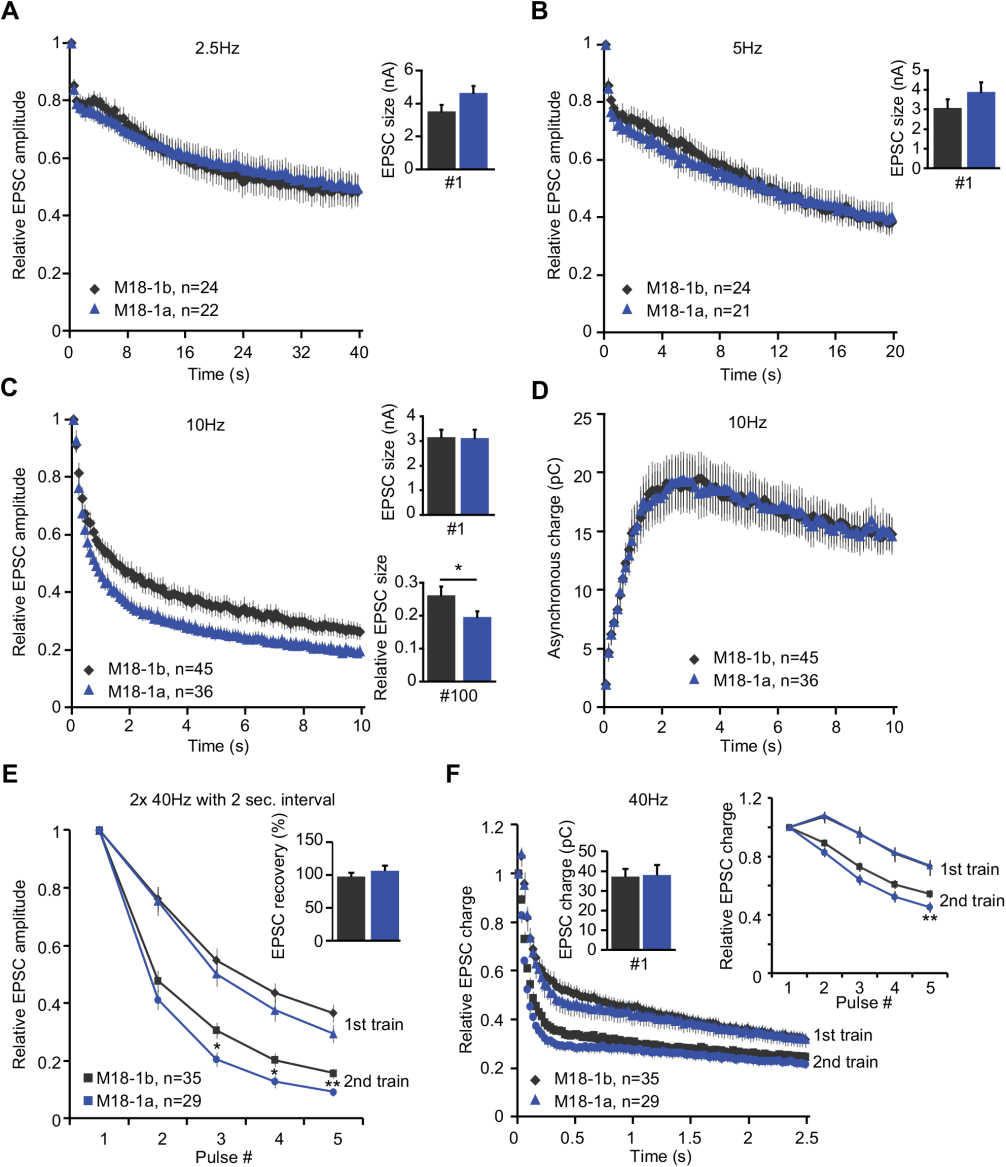
Rate of EPSC depression during STP protocols depends on the splice variant expressed. Munc18-1a or -1b was reintroduced using lentivirus in autaptic hippocampal *munc18-1* null neurons cultured on glia islands. (A) Quantification of EPSC depression during 2.5 Hz stimulation normalized to the first EPSC amplitude (inset shows absolute value of the first EPSC). (B) Quantification of EPSC depression during 5 Hz stimulation normalized to the first EPSC amplitude (inset shows absolute value of the first EPSC). (C) Quantification of EPSC depression during 10 Hz stimulation normalized to the first EPSC amplitude. Left inset shows absolute value of the first EPSC. Right inset shows the relative depression at the end of the train normalized to the first EPSC (M18-1b, 0.26 ± 0.03 EPSC^#100^/EPSC^#1^; M18-1a, 0.20 ± 0.02 EPSC^#100^/EPSC^#1^, Unpaired t test with Welch correction, p = 0.042). (D) Quantification of the build-up of asynchronous charge during 10 Hz stimulation. (E-F) EPSC depression during two stimulation trains (100 pulses at 40Hz) given with a 2 seconds inter-train interval. (E) Shown are the responses to the first five pulses in the train, normalized to the first pulse. Inset shows the recovery of EPSC amplitude after two seconds (1^st^ EPSC of 2^nd^ train / 1^st^ EPSC of 1^st^ train). (F) Quantification of charge transferred within 25ms after each pulse relative to the first pulse in a train. Left inset shows the absolute EPSC charge at the start of the first train, right inset shows the first 5 pulses.

Changes in release probability affect the rate of depression during prolonged stimulation. Therefore, we subjected both groups to stimulation trains of 100 pulses at different frequencies. While the initial EPSC size was similar, the rate of depression during a train depended on the splice variant and the stimulation frequency (Fig 4A–4D). At lower frequencies (2.5 and 5 Hz), the rate of EPSC size depression was equal in neurons expressing Munc18-1b or Munc18-1a (Fig 4A and 4B). At 10 Hz however depression was more pronounced in neurons expressing Munc18-1a (Fig 4C). During high frequency stimulation, synaptic vesicle release becomes less synchronized to individual stimuli due to the rise in global intracellular [Ca^2+^], leading to so-called asynchronous release. The amount of charge transferred in this manner during a 10 Hz train was similar between both groups (Fig 4D). The increased EPSC depression was less pronounced during stimulation at 40 Hz (Fig 4E), potentially due to the fast rate of depression of synchronous release during intense stimulation. EPSC size fully recovered within two seconds after depletion by a 40 Hz stimulation train in both groups, suggesting that alternative splicing of Munc18-1 does not affect synaptic vesicle replenishment (Fig 4E). However, EPSC size depressed faster in neurons expressing Munc18-1a when a second 40 Hz train was given two seconds after the first one (Fig 4F), indicating that Munc18-1a dependent synapses are less capable of sustaining synaptic transmission during bursts of high frequency stimulation. Since asynchronous release quickly becomes the most dominant form of release during a 40 Hz train, the total charge transferred during such a train is also depicted (Fig 4E). Despite initial differences, most likely due to faster depression of synchronous EPSC amplitude in neurons expressing Munc18-1a, both splice variants equally supported release during prolonged stimulation. This rendered the total amount of charge transferred during a 40Hz train equal. These results indicate that Munc18-1a expressing neurons are less efficient in sustaining synchronous release, but not asynchronous release, during high frequency stimulation.

### Munc18-1a is expressed in the Calyx of Held

**Fig 5:**
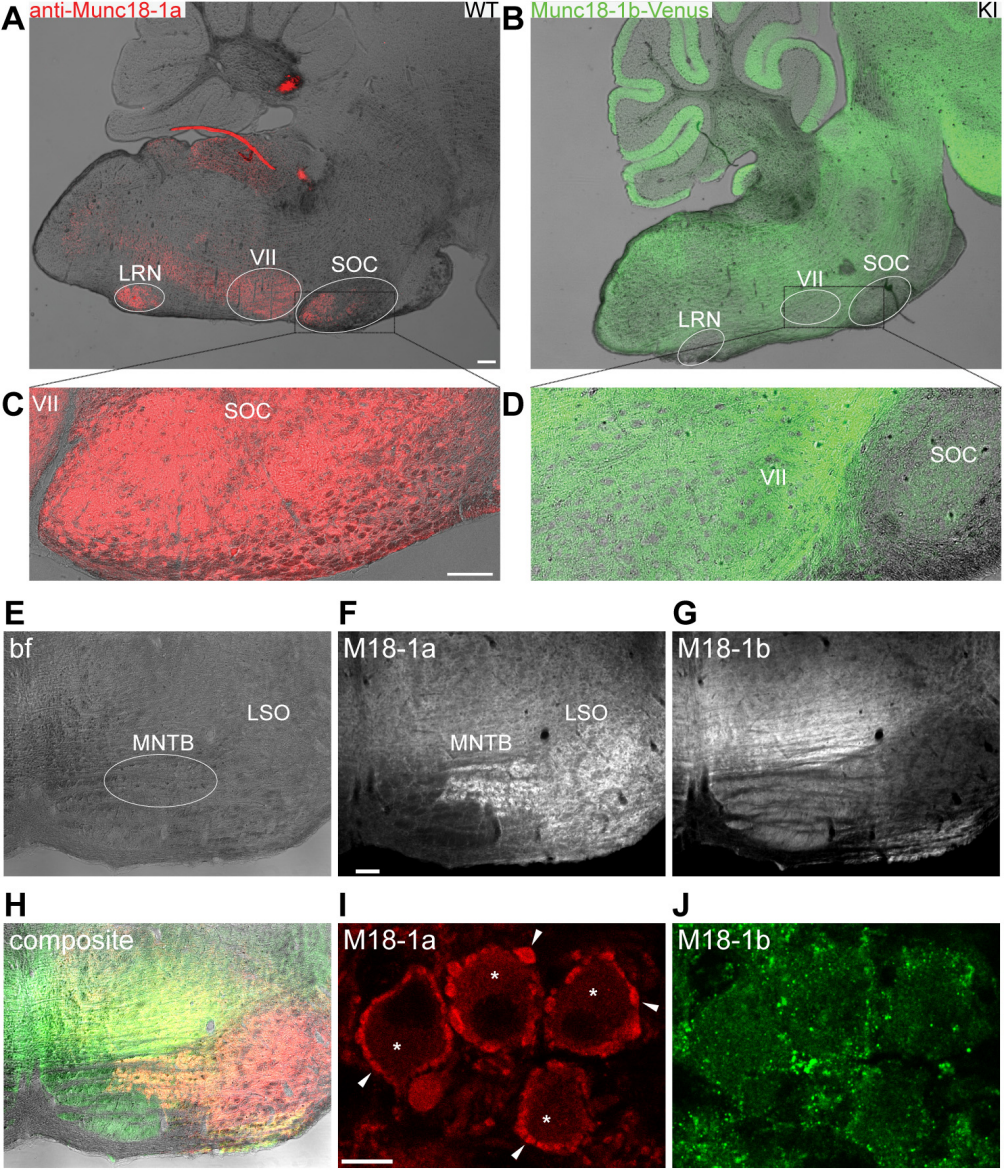
Munc18-1a is expressed in the auditory brainstem of mice. (A) Munc18-1a immunostaining shows specific Munc18-1a expression in the Superior olivary complex (SOC), Facial motor nucleus (VII), and Lateral reticular nucleus (LRN) of the brainstem of wild-type (WT) mice. Scale bar 200 μm. (B) Munc18-1b-Venus is not expressed in nuclei of the brainstem of homozygous Munc18-1-Venus knock-in (M18V KI) mice. (C and D) Higher magnification of indicated regions in A and B, respectively, shows that Munc18-1a is highly expressed in the SOC, which lacks Munc18-1b-Venus expression. Scale bar 100 μm. (E) Bright field (bf) image of a coronal section of P21 wild-type mouse brain (images E-J all from same section), showing part of the brainstem including MNTB (white oval) and LSO. (F) Fluorescence image of immunostaining against Munc18-1a (M18-1a), showing relative high expression in the MNTB and LSO. Scale bar 100 μm. (G) Fluorescence image of immunostaining against Munc18-1b (M18-1b), showing relative low expression in the MNTB and LSO. (H) Composite image of E, F, G showing non-complementary expression of Munc18-1a (red) and Munc18-1b (green). (I and J) Confocal zoom from image in F and G of the MNTB region showing (I) Munc18-1a expression in presynaptic Calyces (arrowheads) and post-synaptic principal cells (*). In contrast, expression of Munc18-1b is punctate but unspecific for Calyces (J). Scale bar 10 μm.

Above results were obtained in neurons from the hippocampus, a region known to mainly express Munc18-1b [10]. To better understand the role of Munc18-1a, we examined in closer detail the expression of Munc18-1 in brainstem, a region reported to have high Munc18-1a expression [10]. We stained for Munc18-1a in wild-type brainstem and compared it to Munc18-1b-Venus expression in M18V KI mice, which were engineered to only express Munc18-1b fused to Venus fluorescent protein (see materials and methods) [17]. We found that Munc18-1a is mainly expressed in specific nuclei of the brainstem: the lateral reticular nucleus (LRN), the facial nucleus (VII), and the superior olivary complex (SOC) (Fig 5A and 5C). Interestingly, these areas, specifically the LRN and the SOC, were mostly devoid of Munc18-1b-Venus (Fig 5B and 5D).

To confirm this complementary expression pattern, we next compared splice variant specific immunostainings within the SOC on coronal section of P21 wild-type mouse brain. Munc18-1a is highly expressed in the medial nucleus of the trapezoid body (MNTB) and lateral superior olive (LSO) (Fig 5E and 5F), while Munc18-1b expression is relatively low/absent in these regions (Fig 5G). Munc18-1a and Munc18-1b are thus expressed complementary within the SOC (Fig 5H). A well-studied auditory synapse in the mammalian central nervous system, the Calyx of Held, is located within this region [26, 27]. The Calyx of Held consists of a presynaptic terminal that engulfs its postsynaptic target, the soma of a principal neuron of the MNTB. Confocal images of the MNTB revealed high expression of Munc18-1a surrounding the large somas of principal neurons (Fig 5I) with a typical Calyx of Held morphology [28–30]. In addition, Munc18-1a expression was also observed in the cytoplasm of principal cells that project to the LSO (Fig 5I), in line with expression in the LSO (Fig 5F). Munc18-1b expression was observed in puncta mostly outside Calyces (Fig 5J) that may be synapses contacting the principal cell other than a Calyx of Held [31, 32]. Hence, Munc18-1a is mainly expressed in specific nuclei of the brainstem, including the LRN, VII and the MNTB and LSO of the superior olivary complex, while Munc18-1b expression is relatively low/absent in these nuclei. The strong specific expression of Munc18-1a in the Calyx of Held within the MNTB suggests that Munc18-1a is the main splice variant in this auditory synapse.

## Discussion

Munc18-1 exists in two alternatively spliced variants, 1a and 1b, which differ at their C-terminus and are expressed at different levels in different brain areas [10]. Splicing factors and their regulators show specific expression profiles with the highest complexity found in the brain [33], increasing transcriptional differences between neuronal cell types. Regulating essential proteins like Munc18-1 by alternative splicing is a potent mechanism to meet cell-type specific requirements for synaptic transmission. While both splice variants supported basal evoked and spontaneous release of synaptic vesicles to a similar extent, neurons expressing Munc18-1b showed less depression of synchronous release during high frequency trains. This suggests that Munc18-1b is more potent in sustaining release during high frequency stimulation.

We found that Munc18-1a and Munc18-1b showed complementary expression in the brainstem, which may suggest specific functions for both splice variants. Our results suggest that Munc18-1a, not the canonical Munc18-1b variant, is the main splice variant expressed in the Calyx of Held. The Calyx of Held synapse generally requires neurotransmission at a higher fidelity and frequency than hippocampal synapses [26]. Several other major components of the synaptic vesicle release machinery show differential isoform expression between hippocampal and Calyx of Held synapses [29, 34, 35]. Subtle changes in the molecular release machinery used by these synapses might explain differences in the efficiency of synaptic transmission.

Munc18-1a neurons showed a trend towards a higher release probability (PPR 20ms interval, Fig 3D) and smaller RRP size (Fig 3C), which could explain the faster EPSC depression during prolonged high frequency stimulation (Fig 4C and 4E). Since hippocampal neurons normally do not express Munc18-1a, at least not at detectable levels, it cannot be excluded that specific conditions/co-factors that would be required for more distinct functional differences, are lacking.

Munc18-1 interacts with the SNARE protein syntaxin1 [36]. Modulation of this interaction is an attractive mechanism to tune synaptic transmission. Both splice variants bind to monomer syntaxin1a [10] and contain the CDK5 phosphorylation site T574 that when phosphorylated reduces the syntaxin-Munc18-1 interaction [37]. However, the posttranslational modification database Phosida [38, 39] and kinase prediction server NetPhos [40] revealed a potential CaMKII site specific for Munc18-1a (S591, arrow in Fig 1A). Although CaMKII is mainly known for its postsynaptic role in LTP upon long-term persistent activation, CaMKII is also activated by short-term calcium elevations [41]. The specific inclusion of a CaMKII phosphorylation site might therefore explain why Munc18-1 splice variants support synchronous release during high-frequency stimulation trains to a different extent. However, CaMKII expression is relatively low during the first two weeks of development and increases in the third and fourth week [42]. Since electrophysiological recordings were made in the third week after culturing, low CaMKII expression might explain why only minor functional differences between Munc18-1 splice variants were observed.

Alternative splicing might also be developmentally regulated. Munc18-1 is involved in neurite outgrowth during early development [43], and this function might be specifically regulated by a single splice variant. In agreement with this hypothesis, autaptic hippocampal neurons rescued with a single splice variant showed differences in morphology, with neurons expressing Munc18-1a showing more synapses and a more complex dendritic tree (Fig 2). The effect of neurite outgrowth on neuronal morphology is only apparent during initial development, but not after synaptogenesis [43]. This might explain why the observed differences in morphology at DIV11 have no impact on synaptic vesicle release at DIV14-18. Alternatively, the morphological differences may have been too mild to affect synaptic transmission.

To summarise, our results suggest that alternative splicing of Munc18-1 generates to two functionally closely related variants of Munc18-1, which differ in their ability to sustain synaptic vesicle release during STP and have differential expression pattern in the auditory brainstem. Alternative splicing of Munc18-1 might thus regulate synaptic plasticity at specific synapses in the brain.
